# Postoperative Quality of Life in Patients Undergoing Surgery for Breast Cancer: A Cross-Sectional Study at a Tertiary Care Hospital of North India

**DOI:** 10.7759/cureus.115843

**Published:** 2026-09-06

**Authors:** Atia Zaka-ur-Rab, Rahul Kumar Keshari, Arun Kengalapura Ramesh Naik, Mohammad Fahud Khurram

**Affiliations:** 1 General Surgery, Jawaharlal Nehru Medical College, Aligarh Muslim University, Aligarh, IND; 2 General Surgery, All India Institute of Medical Sciences, Deoghar, Jharkhand, IND; 3 Cardiac/Thoracic/Vascular Surgery, Atal Bihari Vajpayee Institute of Medical Sciences, Dr. Ram Manohar Lohia Hospital, New Delhi, IND; 4 Plastic and Reconstructive Surgery, Jawaharlal Nehru Medical College, Aligarh Muslim University, Aligarh, IND

**Keywords:** breast cancer, breast-conserving surgery, mastectomy, postoperative outcomes, quality of life

## Abstract

Background: Breast cancer is the most frequently diagnosed cancer among women in India. Due to improvements that have led to higher survival rates, survivorship and quality of life (QoL) have become important goals for care. The aim of the study was to determine the effect of age, stage of the disease, and histopathological type of breast cancer on QoL in patients undergoing surgical intervention for breast cancer.

Methods: This cross-sectional observational study was conducted at a tertiary care institution in North India from 2016 to 2022. This study included 200 women who had definitive surgery (either breast conserving surgery (BCS) or modified radical mastectomy (MRM)) with histological confirmation. QoL was assessed using the European Organisation for Research and Treatment of Cancer Quality of Life Questionnaire BR23 (EORTC QLQ-BR23) across the physical, functional, cognitive, emotional, and social domains.

Result: A total of 200 patients were enrolled in the study. At diagnosis, the mean age was 45.3 ± 12.1 years; 46.5% were premenopausal. Most presented with an advanced stage of disease, with the majority presenting as Stage III (66%). Patients with breast-conserving surgery (10.5%) reported significantly better quality of life than patients with mastectomy in all areas (p < 0.001). Adjuvant radiotherapy was correlated with higher physical (61.6 ± 3.5), cognitive (5.8 ± 0.4), and social (10.5 ± 0.5) scores than non-recipients, with all three showing a significant association (p < 0.001).

Conclusion: The findings emphasize the necessity to integrate QoL assessments into routine oncologic care, especially in resource-limited settings, to improve the overall well-being of breast cancer survivors.

## Introduction

Breast cancer is the most frequently diagnosed cancer among women worldwide and remains the leading cause of cancer-related deaths in this group. In India, it is the most common cancer, accounting for 13.5% of all new cancer cases and 10.6% of all cancer-related deaths, making it a major contributor to the national cancer burden and ranking third among all cancer-related mortalities [1]. Surgical treatments for breast cancer, particularly mastectomy and breast-conserving surgery performed without oncoplastic reconstruction, often result in noticeable disfigurement and loss of breast contour and function. These outcomes profoundly affect a woman’s body image, sexual health, and psychological well-being [2,3]. Body image disturbances following breast cancer surgery can lead to significant psychological distress and are closely associated with increased levels of depression and anxiety. These emotional challenges are often exacerbated by the patient’s inability to adapt to breast disfigurement, resulting in a profound impact on mental health and overall well-being [4-6]. In the context of breast cancer, QoL is multidimensional and includes physical, emotional, social, and functional domains.

As cancer treatments improve and patients live longer, understanding and supporting their quality of life during and after treatment becomes just as important as the treatment itself-especially in advanced stages, where the goal is often to help them feel better, not just live longer [7]. Several reliable tools are available to assess quality of life in cancer patients, and among them, the European Organisation for Research and Treatment of Cancer Quality of Life Questionnaire C30 (EORTC QLQ-C30) questionnaire developed by the European Organization for Research and Treatment of Cancer is one of the most widely used and trusted across different cancer types, especially in clinical trials and research involving patient-reported outcomes [8]. For women with breast cancer, the European Organisation for Research and Treatment of Cancer Quality of Life Questionnaire BR23 (EORTC QLQ-BR23) is used in conjunction with the QLQ-C30, which provides meaningful, disease-specific insights through 23 questions that explore body image, side effects of treatment, sexual well-being, and thoughts about the future, helping to better understand how the illness and its treatment affect their daily lives [9]. Delayed presentation due to lack of awareness about the disease, limited access to psychological support, stigma associated with the disease, mainly in the rural population, and socioeconomic disparities further influence QoL outcomes.

Studies to date on the effect of post-operative QOL in breast cancer survivors have yielded inconsistent results. Moreover, there is a dearth of Indian data on the impact of surgical intervention on the QOL of breast cancer survivors due to the lack of patient-tailored interventions in the socio-cultural context of the country. Given the limited Indian data on breast cancer survivorship and QoL, this study was therefore undertaken to assess current QoL outcomes using the EORTC QLQ-BR23 tool for the identification of domains most affected in post-treatment patients. The findings aim to guide patient-centered interventions tailored to the sociocultural context of our population. The primary aim of the study was to determine the association of quality of life (QOL) with the physical, emotional, cognitive, and social functioning of patients who had undergone surgical intervention as part of their treatment.

## Materials and methods

Study design and setting

This cross-sectional observational study was carried out in the Department of Surgery at Jawaharlal Nehru Medical College, Aligarh Muslim University, Aligarh, India, from October 2016 to January 2022. The study protocol was approved by the Institutional Ethics Committee (IECJNMC/107). The main aim was to assess health-related quality of life (HRQoL) in patients with breast cancer who had received surgical treatment using the European Organisation for Research and Treatment of Cancer Breast Cancer-Specific Quality of Life Questionnaire (EORTC QLQ-BR23) [10].

Study population

Women aged 18 years and older with histologically confirmed primary unilateral breast carcinoma were eligible for inclusion if they underwent definitive surgery (breast-conserving surgery or mastectomy) with clear margins and had no evidence of recurrence or metastasis during follow-up. Patients who failed to complete the prescribed treatment regimen, had distant metastases at the time of evaluation, and had pre-existing psychiatric illness that could interfere with assessment were excluded from the study.

Assessment of quality of life

The European Organisation for Research and Treatment of Cancer Breast Cancer-Specific Quality of Life Questionnaire BR23 (EORTC QLQ-BR23) was used for this assessment [10]. This validated 23-item questionnaire is a module supplementary to the core EORTC QLQ-C30 and evaluates domains including body image, systemic side effects, breast and arm symptoms, sexual functioning, and future perspective. Items are scored on a 4-point Likert scale (1 = Not at all to 4 = Very much) and transformed to a 0-100 scale. For functioning domains, higher scores indicate better functioning; for symptom domains, higher scores denote more severe symptoms. A one-week recall period was used for most items; for sexual functioning, a four-week recall period was applied.

Patients were contacted either in person or via telephone. Data collection occurred six months post-treatment to minimize the influence of acute treatment-related effects. The original English version of the EORTC QLQ-BR23 questionnaire was linguistically validated and translated into Hindi for patients as needed.

Scoring and domain classification

The responses were identified as five major areas: physical, functional, emotional, cognitive, and social well-being. Global and domain-specific scores were calculated as per the EORTC scoring manual. Subgroup analyses were performed by age, menopausal status, tumor stage (TNM classification), histopathological type (invasive ductal carcinoma - not otherwise specified vs. ductal carcinoma in situ), surgical modality (breast-conserving surgery vs. mastectomy), and receipt of adjuvant therapy (chemotherapy and/or radiotherapy).

Statistical analysis

Data were analyzed using IBM SPSS Statistics for Windows, Version 21.0 (IBM Corp., Armonk, NY). Descriptive variables were depicted in the form of numbers and percentages. Continuous variables were summarized using mean and standard deviation. Group comparisons for means of continuous variables were performed using a one-way ANOVA test. A p-value of <0.05 was considered statistically significant.

## Results

This hospital-based cross-sectional study was conducted in the Department of Surgery in collaboration with the Department of Plastic Surgery at Jawaharlal Nehru Medical College, Aligarh Muslim University, Aligarh, from October 2016 to January 2022. The study included 200 patients with biopsy-proven breast carcinoma who had undergone surgical intervention as part of their disease treatment. This study included 21 patients who had undergone Breast Conservative Surgery (BCS) with neoadjuvant chemotherapy and adjuvant radiotherapy and 179 patients who had undergone Modified Radical Mastectomy with neoadjuvant chemotherapy.

The mean age at diagnosis was 45.3 years (SD ±12.1), with the most significant percentage of patients belonging to the 41-50 year age group (30.5%), followed by the 31-40 year age group (27.5%). Almost half of the women (46.5%) were not yet in menopause, and 32% were already in menopause (Table 1). The vast majority of cases, however, were invasive ductal carcinoma-not otherwise specified (IDC-NOS), accounting for 96% of cases, while only 4% were diagnosed with ductal carcinoma in situ (DCIS). Most of the patients presented with advanced disease, with 66% being Stage III (IIIA: 33.5%; IIIB: 32.5%), and 9% presenting with early-stage disease (Stage I-IIA). For surgical management, modified radical mastectomy (MRM) was the primary surgical procedure performed, accounting for 89.5% of cases, while 10.5% underwent breast-conserving surgery (BCS).

**Table 1:** Demographic characteristics of the patients having surgery in breast cancer The data has been represented as N (%).

Characteristics	N(%)
Age	
≤ 30 years	26 (13.0 %)
31 - 40 years	55 (27.5%)
41 - 50 years	61 (30.5%)
51 - 60 years	39 (19.5%)
> 60 years	19 (9.5%)
Menopausal status	
Menopause	43 (21.5%)
Pre-menopause	93 (46.5%)
Postmenopause	64 (32.0%)
Diagnosis	
Ductal carcinoma in-situ	8 (4.0%)
Invasive ductal carcinoma–not otherwise-specified	192 (96.0%)
Stage at diagnosis	
I	5 (2.5%)
IIA	13 (6.5%)
IIB	41 (20.5%)
IIIA	67 (33.5%)
IIIB	65 (32.5%)
IV	9 (4.5%)
Types of operation	
Breast-conserving surgery (BCS)	21 (10.5%)
Mastectomy	179 (89.5%)

Younger patients consistently exhibited significantly greater QOL scores across domains (p < 0.05). Women aged ≤30 years had the highest QOL scores among all subgroups, with mean scores of physical (53.5 ± 8.4), emotional (23.1 ± 4.7), and social (8.7 ± 2.3) functioning, while values were lowest for women aged 51-60 years and >60 years. Emotional well-being declined steadily with age, with the lowest quality-of-life score for women >60 years (18.5 ± 3.1). Menopausal status was also a significant predictor of QOL. Premenopausal women had better physical (49.7 ± 7.1), functional (12.7 ± 2.0), and emotional (20.9 ± 3.8) QOL than either menopausal or postmenopausal women, p < 0.05. Although cognitive scores were not statistically different (p = 0.194) between the menopausal groups, social functioning scores were highest among premenopausal women (8.0 ± 1.9) (Table 2).

**Table 2:** QOL according to age and menopausal status The data has been represented as mean ± SD. For significance, a p-value of less than 0.05 was considered significant. The significant values are marked with *.

Characteristics	Physical (mean ± SD)	Functional (mean ± SD)	Cognitive (mean ± SD)	Emotional (mean ± SD)	Social (mean ± SD)
Age					
≤ 30 years	53.54 ± 8.4	13.77 ± 2.1	4.77 ± 1.3	23.12 ± 4.7	8.69 ± 2.3
31 - 40 years	48.18 ± 6.2	12.20 ± 1.9	3.98 ± 1.2	19.89 ± 2.8	7.69 ± 1.8
41 - 50 years	46.21 ± 4.1	12.05 ± 1.3	4.11 ± 0.9	19.61 ± 3.4	7.43 ± 1.4
51 - 60 years	45.69 ± 3.6	11.82 ± 1.3	3.97 ± 0.7	19.95 ± 3.5	7.08 ± 1.4
> 60 years	46.95 ± 3.7	12.00 ± 1.7	4.11 ± 0.9	18.47 ± 3.1	6.89 ± 1.4
p-value	F = 10.50; p = 0.0001*	F = 6.62; p= 0.000*	F = 3.12; p= 0.021*	F = 6.45; p= 0.000*	F = 4.80; p = 0.001*
Menopausal status					
Menopause	45.77 ± 3.6	11.86 ± 1.3	3.91 ± 0.9	19.65 ± 3.1	7.37 ± 1.4
Pre-menopause	49.68 ± 7.1	12.65 ± 2.0	4.26 ± 1.2	20.91 ± 3.8	7.99 ± 1.9
Postmenopause	46.05 ± 3.6	11.98 ± 1.4	4.11 ± 0.8	19.22 ± 3.5	7.02 ± 1.5
p-value	F = 11.48; p= 0.000*	F = 4.56; p = 0.018	F = 1.75; p= 0.194*	F = 4.69; p = 0.011*	F = 6.62; p= 0.002*

Quality of life scores steadily decreased with advancing stage of breast cancer in all functional domains (p < 0.001) (Table 3). Patients with Stage I disease reported the highest scores related to physical functioning (61.8 ± 2.6), role functioning (16.0 ± 1.0), cognitive function (5.8 ± 0.4), emotional well-being (25.6 ± 3.2), and social functioning (10.2 ± 0.4). Patients with Stage IV disease reported the lowest scores, with mean scores of emotional functioning (18.3 ± 2.1) and physical functioning (44.8 ± 2.1). Patients with intermediate disease (IIA-IIIB) had a gradual decline across all domains. Patients with Stage III disease had significantly lower functional and cognitive scores compared to patients diagnosed with early-stage disease.

**Table 3:** QOL by stage of disease The data has been represented as mean ± SD. For significance, a p-value of less than 0.05 was considered significant. The significant values are marked with *.

Stage	Physical (mean ± SD)	Functional (mean ± SD)	Cognitive (mean ± SD)	Emotional (mean ± SD)	Social (mean ± SD)
I	61.80 ± 2.6	16.00 ± 1.00	5.80 ± 0.4	25.60 ± 3.2	10.20 ± 0.4
IIA	57.54 ± 8.3	14.69 ± 2.0	5.38 ± 0.9	25.15 ± 3.6	9.92 ± 1.3
IIB	48.63 ± 6.4	12.49 ± 1.6	4.32 ± 1.0	20.88 ± 4.3	7.59 ± 1.8
IIIA	45.81 ± 3.6	11.66 ± 1.4	3.94 ± 0.8	19.01 ± 2.4	7.21 ± 1.5
IIIB	46.34 ± 3.1	12.02 ± 1.5	3.80 ± 1.0	19.54 ± 3.2	7.23 ± 1.5
IV	44.78 ± 2.1	12.00 ± 1.1	4.44 ± 0.5	18.33 ± 2.1	7.22 ± 1.2
p-value	F = 26.44; p= 0.000*	F = 15.73; p = 0.000*	F = 11.28; p = 0.000*	F = 12.29; p = 0.000*	F = 10.55; p = 0.000*

Patients with ductal carcinoma in situ (DCIS) had slightly higher cognitive scores (4.25 ± 0.8) than those with invasive ductal carcinoma-not otherwise specified (IDC-NOS), while the IDC-NOS cohort had significantly higher emotional (20.19 ± 3.6 vs. 18.00 ± 3.3; p = 0.01) and social functioning scores (7.57 ± 1.7 vs. 7.00 ± 1.4; p = 0.037) (Table 4). In terms of surgical approach, breast-conserving surgery (BCS) had superior outcomes across all domains. Overall, patients treated with BCS had significantly higher physical (61.57 ± 3.5 vs. 46.04 ± 3.4; p < 0.001), emotional (26.05 ± 2.7 vs. 19.40 ± 3.1; p < 0.001), and social functioning scores (10.48 ± 0.05 vs. 7.20 ± 1.5; p < 0.001) compared to those with mastectomy. Similarly, adjuvant radiotherapy increased improvements in quality of life in each domain. Patients receiving radiotherapy had improved physical (61.57 ± 3.5), cognitive (5.81 ± 0.40), and social scores (10.48 ± 0.5) compared to patients receiving no radiotherapy intervention (p < 0.001).

**Table 4:** QOL by histopathological subtype, surgery and radiotherapy The data has been represented as mean ± SD. For significance, a p-value of less than 0.05 was considered significant. The significant values are marked with *.

Subtype	Physical (mean ± SD)	Functional (mean ± SD)	Cognitive (mean ± SD)	Emotional (mean ± SD)	Social (mean ± SD)
DCIS	46.00 ± 1.6	12.25 ± 1.1	4.25 ± 0.8	18.00 ± 3.30	7.00 ± 1.4
IDC-NOS	47.74 ± 5.9	12.27 ± 1.8	4.13 ± 1.0	20.19 ± 3.6	7.57 ± 1.7
P value	F = 0.69; p = 0.0411*	F = 0.001; p = 0.981	F = 0.11; p = 0.755	F = 2.86; p = 0.01*	F = 0.87; p = 0.037*
Operation					
BCS	61.57 ± 3.5	15.57 ± 0.8	5.81 ± 0.4	26.05 ± 2.7	10.48 ± 0.5
Mastectomy	46.04 ± 3.4	11.88 ± 1.4	3.94 ± 0.9	19.40 ± 3.1	7.20 ± 1.5
P value	F = 389.78; p = 0.000*	F = 140.10; p = 0.000*	F = 88.30; p = 0.000*	F = 88.65; p = 0.000*	F = 98.73; p = 0.000*
Adjuvant radiotherapy					
Not Received	46.04 ± 3.4	11.88 ± 1.4	3.94 ± 0.9	19.40 ± 3.1	7.20 ± 1.5
Received	61.57 ± 3.5	15.57 ± 0.8	5.81 ± 0.4	26.05 ± 2.7	10.48 ± 0.5
p-value	F = 389.78; p= 0.000*	F = 140.10; p = 0.000*	F = 88.30; p = 0.000*	F = 88.65; p = 0.000*	F = 98.73; p = 0.000*

## Discussion

Breast cancer treatment today emphasizes not just survival but also survivorship and quality of life (QoL). Our study highlights how factors such as age, menopausal status, stage of disease, surgical approach, and adjuvant therapies independently shape the postoperative QoL of Indian women. Our study supported the assertion that multiple factors-age, menopausal status, cancer stage, surgical approach, adjuvant therapy, and histological type-independently influence QOL. Kaur et al. (2018) in North India assessed the prevalence of post-treatment issues such as fatigue, joint pain, shoulder stiffness, chemotherapy-induced amenorrhea, and loss of libido. They concluded that although QoL tends to improve over time, it remains poorer compared to healthy counterparts, with emotional distress, fatigue, and premature menopause having the most substantial impact [11].

Dubashi et al. (2010) showed that younger survivors (age < 35 years) had lower sexual functioning despite having excellent physical and functional scores. Pain and arm morbidity were higher among mastectomy patients, while patients undergoing breast-conserving surgery had better outcomes [12]. Sharma et al. (2017) correlated QoL scores with disease status at 18 months, finding the worst outcomes among patients with metastatic disease [13]. Disease-free patients had significantly higher global QoL and functioning scores. In our study, we found that young, premenopausal women diagnosed early and treated with BCS and adjuvant radiotherapy had the best QoL outcomes. On the other hand, older, postmenopausal patients with advanced-stage disease who had a mastectomy without any additional treatment had the worst quality of life.

In low-resource settings, patient-related factors such as delayed hospital visits, sociocultural stigma, low awareness, and economic constraints further aggravate QoL impairment. Sharma et al. (2012) found that lower education levels and poor income significantly delayed treatment [14]. This evidence suggests that enhancing early detection, patient support, and access to affordable care can significantly improve post-treatment QoL.

Younger women (≤30 years) and premenopausal patients in our cohort reported significantly better physical, emotional, cognitive, and social functioning scores. These findings are consistent with Imran et al., who reported superior QoL among younger breast cancer survivors using the EORTC QLQ-C30 and BR23 questionnaires [15]. Similarly, Gangane et al. observed that postmenopausal women and those with limited social support were more likely to have lower emotional health scores [16]. This suggests that hormonal changes and psychosocial adaptability are critical determinants of QoL. Patients with early-stage disease (Stage I-IIA) had significantly higher QoL scores across all domains compared to those with advanced stages. Prior studies support this trend, as advanced disease is associated with fatigue, pain, and emotional distress [17]. Sharma et al. (2017) also noted that metastatic patients had the poorest QoL, while disease-free survivors reported the highest scores [13]. These findings emphasize the value of early detection and treatment in improving survivorship outcomes.

Our analysis indicated that women undergoing breast-conserving surgery (BCS) reported significantly better QoL than those treated with modified radical mastectomy (MRM). This agrees with Akca et al. [18] and Enien et al. [19], who demonstrated that BCS provides better outcomes in terms of body image, functional status, and psychological well-being. Kim et al. also highlighted that cosmetic satisfaction after BCS strongly influences long-term QoL [20]. Conversely, mastectomy was linked to body image disturbances and greater psychosocial stress, as described in both Indian and Western literature. Adjuvant radiotherapy in our study was associated with significantly higher physical, cognitive, and social functioning scores. Hartl et al. similarly reported improved psychological recovery in women receiving radiotherapy after BCS compared with those undergoing mastectomy alone [21]. Dubashi et al. noted that while mastectomy patients experienced higher arm morbidity, those receiving BCS and radiotherapy had better overall QoL outcomes [12]. Thus, adjuvant therapy not only improves oncologic safety but also enhances long-term survivorship.

Patients with ductal carcinoma in situ (DCIS) showed better emotional and cognitive outcomes than those with invasive ductal carcinoma (IDC). While literature on histological subtypes is limited, some evidence suggests that DCIS patients perceive their illness as less threatening, resulting in better psychological adjustment [22]. Nevertheless, both subgroups demonstrated significant emotional burden, necessitating continued counseling and psychosocial care.

This study has certain limitations. The cross-sectional design precludes the establishment of causal relationships between patient characteristics, treatment-related factors, and quality-of-life outcomes. As the study was conducted at a single tertiary care center in North India, it limits the generalizability of the findings to other populations and healthcare settings. Furthermore, the absence of a pre-operative baseline quality-of-life assessment restricted the evaluation of changes in quality of life attributable to surgery and adjuvant treatments over time. Finally, the relatively small number of patients who underwent breast-conserving surgery compared with modified radical mastectomy may have influenced comparisons between surgical modalities. The confounding factors could also not be accounted for in the absence of multivariate analysis. Despite these limitations, the research determines the determinants of post-operative quality of life among breast cancer survivors in a resource-limited setting and contributes to the limited evidence available from India. Authorization for academic use of the EORTC QLQ-BR23 questionnaire was obtained through the EORTC Quality of Life Group. The questionnaire was originally developed and validated by Sprangers et al. [10].

## Conclusions

This study concluded that the postoperative quality of life of Indian breast cancer survivors is influenced by age, menopause status, disease stage, surgical type, and adjuvant radiotherapy. Younger, premenopausal women with early disease stages receiving a breast-conserving surgical procedure and radiotherapy reported the best postoperative outcomes. Older, postmenopausal women diagnosed with an advanced disease stage and undergoing mastectomy reported the worst. It is therefore important for healthcare providers to consider the survivor's quality of life as an outcome. Although a causal relationship cannot be established owing to the study design, quality-of-life findings will help modify survivorship care to be culturally relevant for women participating in the study.
